# Identification of a six-lncRNA signature based on a competing endogenous RNA network for predicting the risk of tumour recurrence in bladder cancer patients

**DOI:** 10.7150/jca.35801

**Published:** 2020-01-01

**Authors:** Danfeng Zhao, Qiang Peng, Lu Wang, Cong Li, Yulin Lv, Yong Liu, Zhichao Wang, Ruizhe Fang, Jiaqi Wang, Zhongqing Liu, Wanhai Xu

**Affiliations:** 1 Department of Urology, the Fourth Hospital of Harbin Medical University, Harbin Medical University, Harbin, P. R. China.; 2 Heilongjiang Key Laboratory of Scientific Research in Urology, Harbin, P. R. China.; 3 Department of Urology, Qitaihe People's Hospital, Qitaihe, P.R. China.

**Keywords:** Bladder cancer, Recurrence risk, Recurrence free survival, LncRNA, ceRNA network

## Abstract

Bladder cancer (BC) is the most common malignancy involving the urinary system, and is characterized by a high recurrence rate. It is important to identify potential lncRNA signatures capable of predicting tumour recurrence risk and assessing recurrence prognosis in BC patients. We extracted data from The Cancer Genome Atlas and identified 381 differentially expressed lncRNAs, 855 mRNAs and 70 miRNAs between non-recurrent and recurrent BC tissues. Subsequently, a competing endogenous RNA (ceRNA) network composed of 29 lncRNAs, 13 miRNAs and 4 mRNAs was established. We used univariate and multivariate Cox regression to analyse the relationship between the 29 lncRNAs and recurrence-free survival (RFS) in BC patients. Six lncRNAs had significant prognostic values, and their cumulative risk score indicated that this 6-lncRNA signature independently predicted RFS in BC patients. We applied a receiver operating characteristic (ROC) analysis to assess the efficiency of our prognostic models. High-risk patients exhibited a poorer prognosis than low-risk patients did. Additionally, the 6-lncRNA signature showed a significant correlation with BC clinicopathological characteristics, which indicates that it could be used for effective risk stratification. The current study provides novel insights into the lncRNA-related ceRNA network and this 6-lncRNA signature may be an independent prognostic factor in predicting the recurrence of BC patients.

## Introduction

Bladder cancer (BC) is the most common malignancy involving the urinary system and the ninth most common malignancy worldwide, with an estimated 429,000 new cases and 165,000 deaths per year in the world 1-2. In patients with non-muscle-invasive bladder cancer, the risk of recurrence after 5 years ranges from 50% to 70% 3, for locally advanced bladder cancer, which has a 5-year rate of pelvic recurrence after radical cystectomy as high as 20-45% 4. The high recurrence rate is a prominent feature of bladder cancer 5, and on a per patient basis, bladder cancer is the most expensive solid tumour type 6. Surveillance for bladder cancer is important because of the high rate of recurrence of both non-muscle-invasive and muscle-invasive disease and the short time to progression and death in patients with metastatic disease. Cystoscopy and cytology are currently the standard modalities used to monitor urothelial carcinoma. Cystoscopy can provide high diagnostic accuracy, but it is invasive and often inconvenient, making it impractical for mass screening of bladder cancer in people without signs or symptoms 3; voided urine cytology, as the most widely used non-invasive approach, has high specificity for detection and monitoring of BC, but lacks the sensitivity necessary to rule out cancer 7. Despite the benefit in the prevention of recurrence, there is still no great progress in preventing recurrence. Therefore, it is urgent to discover new biomarkers with high sensitivity and specificity for recurrence prediction of BC that can play pivotal roles in improving the prognosis of BC patients.

Long noncoding RNAs (lncRNAs) are tentatively defined as noncoding RNAs that are more than 200 nucleotides in length but lack protein coding capacity 8. The roles of lncRNAs in human cancers have received considerable attention in various types of cancers, including BC 9. Growing evidences have shown that lncRNAs are abnormally expressed in cancer tissues and typically exhibit tissue-specific expression patterns, which plays an important role in tumourigenesis 10-11. Moreover, lncRNA expression may confer clinical information about disease outcomes and have utility as a biomarker in diagnosis and prognostication 12. Thus, the identification of a BC specific lncRNA biomarker may be of clinical significance for risk stratification and recurrence prediction, which would be valuable for improving the management of BC patients. Regarding BC, several lncRNAs have been investigated in vivo and in vitro experiments, some of which 13-14 even exhibited correlations with clinicopathologic parameters and survival outcomes. Currently, some lncRNAs were detected in the serum or urine of BC patients, although those lncRNAs have been found to predict the clinical outcome of BC. The results have been mainly concluded from single-centre research studies with a relatively small number of BC patients 14-15. Furthermore, studies without a large sample size are also not able to make determinations with statistical power; thus, we used The Cancer Genome Atlas (TCGA) to obtain a large number of BC samples that can provide multidimensional molecular profiles of BC-specific lncRNAs.

Salmenta et al. 16 presented the competing endogenous RNA (ceRNA) hypothesis whereby a novel regulatory mechanism was introduced between non-coding (ncRNA) and coding messenger RNA (mRNA). RNA transcription components can communicate with each other via miRNA response elements (MREs). lncRNAs contain MREs that function as ceRNAs, and play a key role in various pathological processes 17. Their aberrant expression disrupts miRNA-mediated lncRNA/mRNA ceRNA crosstalk interactions, contributing to the initiation and development of cancer 16,18. lncRNA-miRNA-mRNA ceRNA networks have been identified in many types of cancer 19-21. However, to the best of our knowledge, there are no comprehensive analyses of BC recurrence-associated lncRNAs based on a ceRNA network in the context of a large sample size.

In this study, we focused on ceRNA networks to provide a novel perspective and insight into BC and suggested that the signature based on six lncRNAs may be particularly useful for recurrence surveillance, and can serve as an independent prognostic factor for recurrence of BC patients. It will be helpful to identify bladder cancer patients with a higher risk of recurrence and will improve screening and targeted molecular therapeutic approaches for recurrence prevention.

## Methods

### Patients and samples from the TCGA database

RNA sequencing (RNA-Seq) data associated with BC were retrieved from the TCGA database. Information on 409 patients was extracted from TCGA. The exclusion criteria were (1) histological diagnosis negating BC, (2) other malignancy aside from BC, and (3) lack of complete clinical data. In addition to the RNA expression data, clinical data such as pathologic stage and TNM information were also downloaded. Ultimately, data for 244 non-recurrent tumour tissue samples and 119 recurrent tumour tissue samples were analysed. No approval from the ethics committee was needed because all the information was required from the TCGA database.

### RNA sequence data processing and differential expression analysis

All expression profiles were normalized within and among samples. To identify potential RNAs involved in the development of BC, the differentially expressed mRNAs (DEmRNAs), lncRNAs (DElncRNAs), and miRNAs (DEmiRNAs) between non-recurrent and recurrent BC tissues were analysed using the EdgeR package in R, and the cut-off criteria were set as P<0.05 and |logFC|>2.0. Volcano plots were visualized using the ggplot2 packages in R. The heat map was plotted using the pheatmap function of pheatmap package version 1.0.8.

### Functional enrichment analysis

To understand the underlying biological roles and pathways between differentially expressed genes in the ceRNA network. We use DAVID 6.8 (Database for Annotation, Visualization, and Integrated Discovery, https://david.ncifcrf.gov/) for functional enrichment analysis. Fisher's test was used to identify the significant Gene Ontology (GO) terms, and GO categories with P < 0.05 were considered statistically significant. Kyoto Encyclopedia of Genes and Genomes (KEGG) were searched for pathways at the significance level set (P < 0.05 and enrichment score >1.5).

### Construction of the ceRNA network

The ceRNA network was constructed based on the theory that lncRNAs can affect miRNA and act as miRNA sponges to further regulate mRNA 16. Based on this hypothesis, we established the lncRNA-miRNA-mRNA ceRNA network in the following steps: (1) according to the miRcode database (http://www.mircode.org), differentially expressed lncRNAs were used to pair differentially expressed miRNAs. (2) the paired miRNAs were used to identify target mRNAs according to three databases: miRDB (http://www.mirdb.org/), miRTarBase (http://mirtarbase.mbc.nctu.edu.tw/) and TargetScan (http://www.targetscan.org/) programs. Only mRNAs predicted by all three databases were defined as target mRNAs. We ultimately retained intersections with the differentially expressed lncRNAs, miRNA, and mRNAs. Cytoscape (version 3.6.1) was used to visualize the lncRNA-miRNA-mRNA ceRNA network.

### Construction of the BC‑specific recurrent signatures based on the ceRNA network

We further performed recurrence prognostic analyses of the 29 lncRNAs, 13 miRNAs, and 4 mRNAs in the ceRNA network. Univariate Cox proportional hazards regression analysis was used to analyse the relationship between the DElncRNAs and RFS based on the cut-off of p < 0.05 by the R survival package. Next, a multivariate Cox proportional hazards regression model was constructed to predict the risk score of each patient based on the expression of lncRNA. The risk assessment score for predicting recurrence free survival was calculated as follows: Risk score = exp_lncRNA1_ ∗ β_lncRNA1_ + exp_lncRNA2_ ∗ β_lncRNA2_+ exp_lncRNAn_ ∗ β_lncRNAn_ (where “exp” denotes the expression level of DElncRNAs, and “β” is the regression coefficient obtained from the multivariate Cox regression model). Based on the risk score, BC patients were divided into two groups: “low-risk” and “high-risk” group. A Kaplan-Meier curve analysis was conducted to compare the recurrence times of the low-risk group and high-risk group, P < 0.05 was considered statistically significant. The chi-square test was utilized to correlate risk level with clinical parameters including age, gender, T stage, N stage, M stage, Grade stage, pathological stage. Time-dependent ROC curve was performed to assessment the efficiency of our prognostic models. In addition, univariate and multivariate analyses were used to evaluate the effects of clinical characteristics and risk score on the RFS of BC patients. The R software (version 3.5.2) was used for all statistical analyses.

### Cell culture and transfection

Bladder cancer cell lines were obtained from American Type Culture Collection (ATCC). DMEM containing 10% FBS was used to culture cells at 37℃ in 5% CO2. si‐STEAP3‐AS1 vectors were synthesized by RiboBio (China, Guangzhou) and then transfected into cells using Lipofectamine. GAPDH was used as an endogenous control to normalize lncRNA and mRNA, while U6 was used for miRNA. The relative expression level was calculated by 2-ΔΔCt.

## Results

### Identification of DEmRNAs, DElncRNAs, and DEmiRNAs

**Figure 1:**
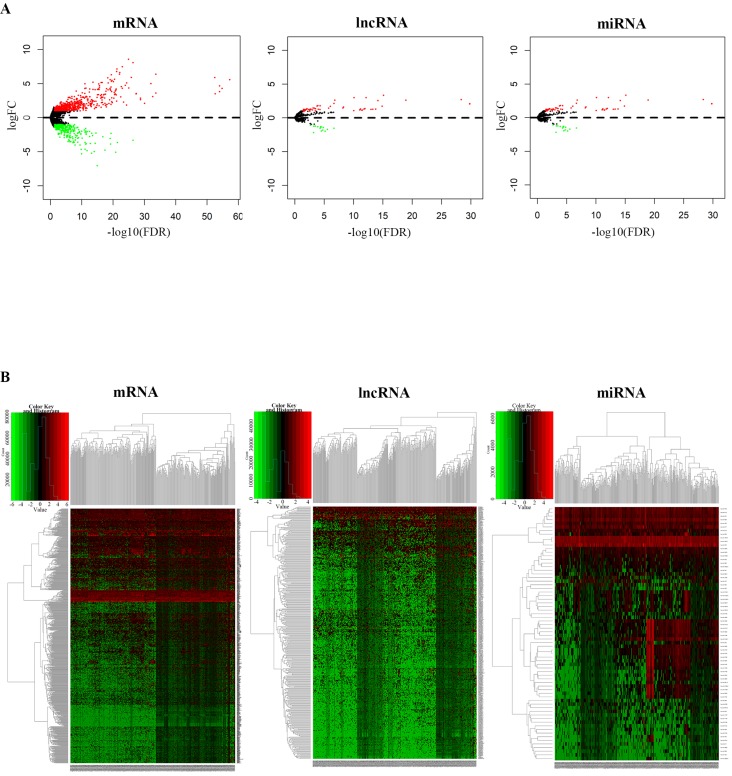
Differential expression of RNAs between non-recurrent and recurrent BC tissues. **A,** Volcano plots showing the differential expression of RNAs (mRNAs, lncRNAs, and miRNAs) and **B,** heatmaps demonstrate differential expression of RNAs (mRNAs, lncRNAs, and miRNAs).

We identified the DEmRNAs, DElncRNAs and DEmiRNAs in non-recurrent and recurrent BC tissues using the TCGA database. Differentially expressed RNAs were analysed using the edgeR package following extraction of the expression matrix from 363 BC cases, using p<0.05 and |logFC|>2.0 as the cut-off criteria. A total of 381 differentially expressed lncRNAs (250 upregulated and 131 downregulated), 70 differentially expressed miRNAs (55 upregulated and 15 downregulated), and 855 differentially expressed mRNAs (566 upregulated and 289 downregulated) were identified by comparing non-recurrent and recurrent BC tissues. Volcano plots displayed the distribution of the DEmRNAs, DElncRNAs and DEmiRNAs (Fig. 1A). The heat map showed clear separation and consistency in the expression profiles of the non-recurrent and recurrent BC tissues (Fig. 1B).

### Enrichment Analysis of Gene Ontology and KEGG Pathways

**Figure 2:**
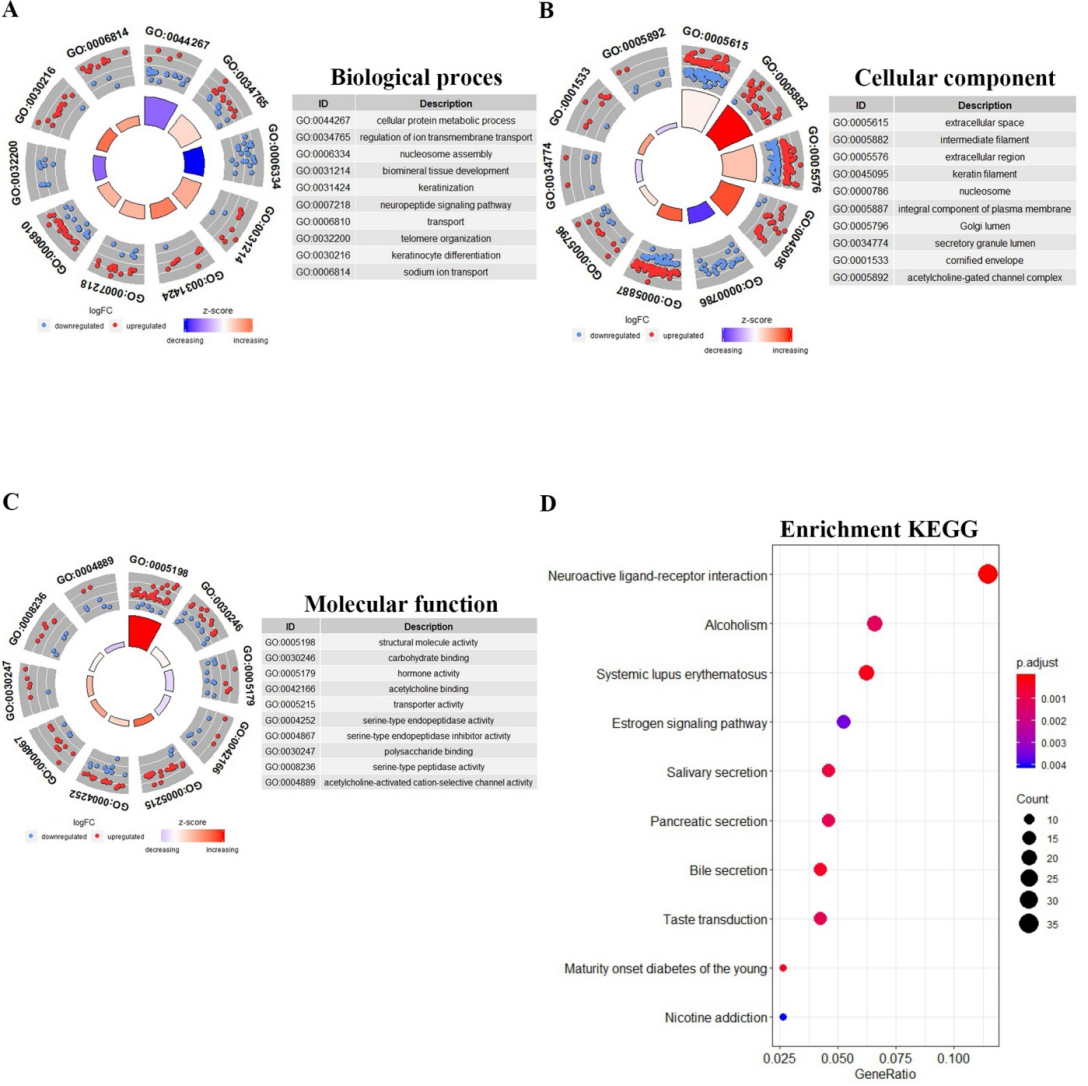
Gene Ontology and KEGG pathways enrichment analysis of differentially expressed mRNAs. The 10 most significantly GO results covering domains of **A,** biological processes (BP), **B,** cellular component (CC) and **C,** molecular function (MF). **D,** The 10 most significantly enriched KEGG pathways.

For further study of the functions of the differentially expressed genes, a total of 855 differentially expressed mRNAs were analysed and the top 10 GO results and 10 KEGG pathways are shown. The Goplot package of R was utilized to compare gene clusters based on their enriched biological processes with a cutoff of p < 0.05. In the biological processes (BP), the target genes were significantly clustered into items including cellular protein metabolic process, regulation of ion transmembrane transport, nucleosome assembly, and keratinization (Fig. 2A). Meanwhile, we found that the mRNAs were highly associated to cellular component (CC), including extracellular space, intermediate filaments, extracellular region, keratin filament, and nucleosome (Fig. 2B). Structural molecule activity, carbohydrate binding, hormone activity and other vital molecular function (MF) were significantly related to these genes (P-value < 0.05) (Fig. 2C). The GO enrichment networks of BP, CC and MF for these genes were filtered for GO terms with P<0.05 and Benjaminni corrected P<0.05. In the KEGG analysis, these mRNAs were mainly enriched in neuroactive ligand-receptor interaction, alcoholism, systemic lupus erythematosus, and estrogen signaling pathway. The 10 most significant KEGG pathways are shown in Fig. 2D.

### Construction of the ceRNA network based on predicted miRNA targets

**Figure 3:**
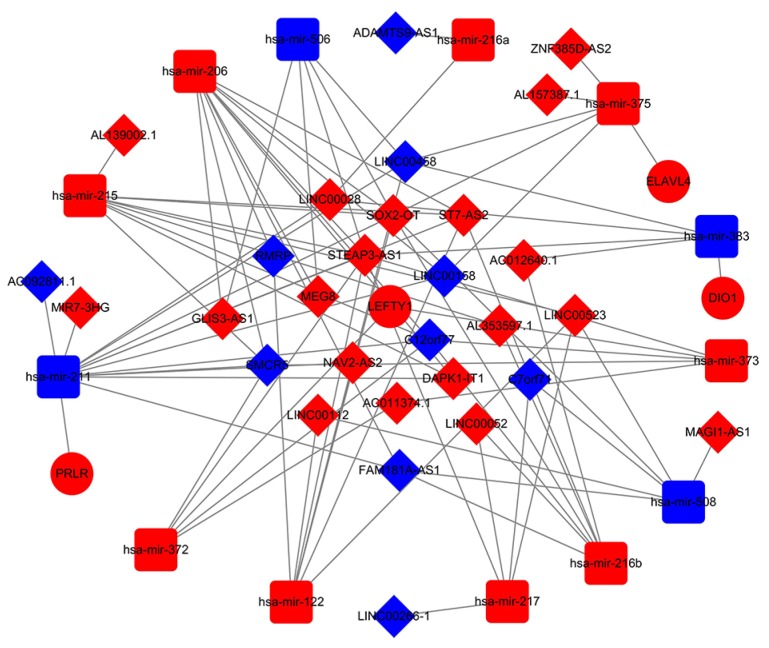
The ceRNA network of lncRNA-miRNA-mRNA. In network the blue nodes indicate down-regulated expression, and the red nodes indicate up-regulated expression. Rectangles represent miRNAs, ellipses represent protein-coding genes, and diamonds represent lncRNAs; gray edges indicate lncRNA-miRNA-mRNA interactions.

Numerous studies have suggested that lncRNAs interact with miRNA response elements to act as miRNA sponges 16. A ceRNA network was constructed based on the lists of DElncRNA, DEmiRNA, and DEmRNA. First, the target regulation network of lncRNA-miRNA was evaluated. A total of 83 potential lncRNA-miRNA pairs, including 29 lncRNAs and 13 miRNAs, were identified based on miRcode (Additional file, Table S1). Next, the MiRDB, miRTarBase and Targetscan programmes were used to predict the mRNA targets of the miRNAs. In total, 5 miRNA-mRNA pairs were identified, including 4 mRNAs and 5 miRNAs (Additional file, Table S2). Based on above information, we constructed a lncRNA-miRNA-mRNA ceRNA network using Cytoscape 3.6.1 including 13 miRNAs (Table 1), 4 mRNAs (Table 2), and 29 lncRNAs (Table 3) were involved in the ceRNA network. Cytoscape software (version 3.6.1) was used to visualize the lncRNA-miRNA-mRNA ceRNA network (Fig 3).

### Construction of a recurrent signature based on the ceRNA network

**Figure 4:**
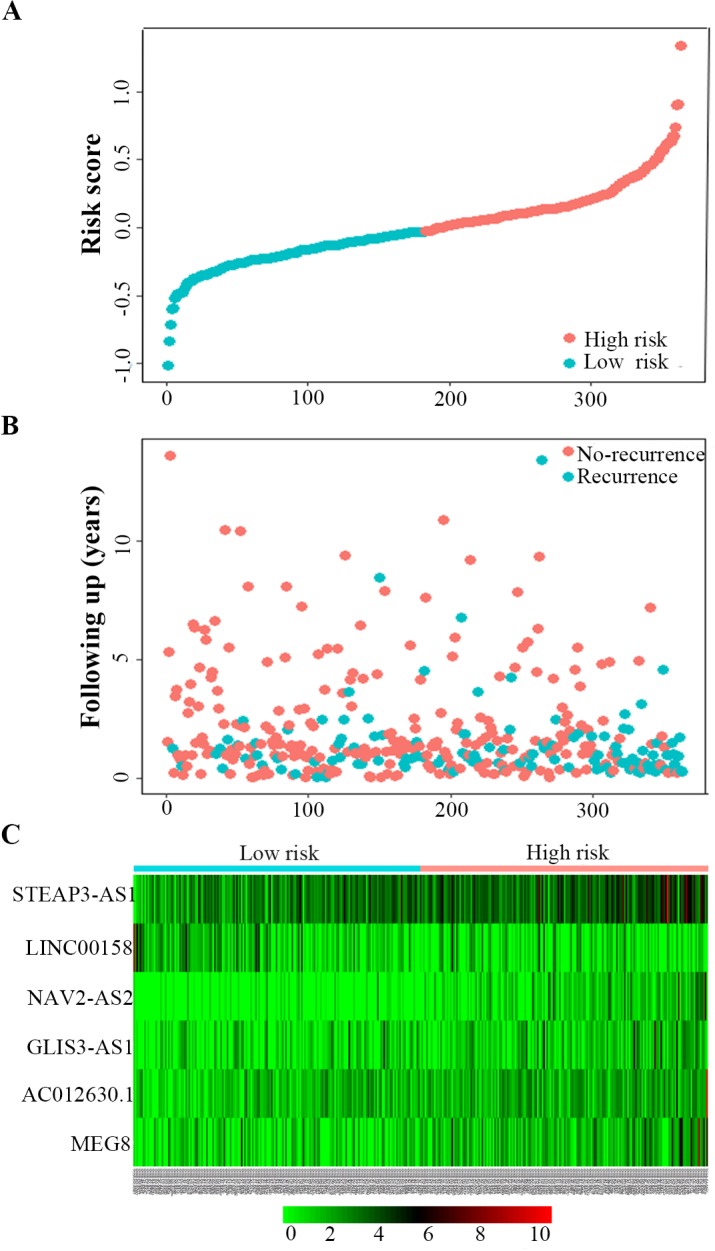
Six-lncRNA signature predicted RFS in bladder cancer patients. Kaplan-Meier survival curves of RFS between high-risk and low-risk patients, the distributions of **A,** patients' risk score **B,** recurrence status and **C,** heat map of the six-lncRNA expression profiles in low- and high-risk patients.

Univariate Cox regression analysis was used to identify the lncRNAs associated with the RFS of BC patients. In total, 9 lncRNAs were detected to have significant recurrent prognostic value, with the significance level cut-off threshold set at P < 0.05 (Additional file, Table S3). All of the above lncRNAs were further submitted to multivariate Cox regression analysis to identify the independent prognostic predictors for BC, which indicated that only six lncRNAs—MEG8, NAV2-AS2, STEAP3-AS1, GLIS3-AS1, LINC00158, AC012640.1—had an independent prognostic value in BC, and these six lncRNAs were used to develop a lncRNA recurrent prognostic model. Next, the risk scores for predicting recurrence were constructed based on the six lncRNAs. The risk assessment score for predicting RFS was calculated as follows: Risk score= [expression value of AC012640.1 × 0.247] + [expression value of STEAP3-AS1 × 0.225] + [expression value of NAV2-AS2 × 0.296] + [expression value of MEG8 × 0.174] + [expression value of GLIS3-AS1 × 0.127] + [expression value of LINC00158 × -0.184]. As shown in Fig. 4, the scores assigned to each patient provide a good assessment of recurrence. Based on the risk score, bladder cancer patients were divided into two groups: “low-risk” and “high-risk” groups (Fig 4A). The recurrence rate of the high-risk patients was significantly higher compared to the low-risk patients (44.8% vs 20.9%; P < 0.01) (Fig 4B). The gene expression profiles of the 6-lncRNA signature in high-risk and low-risk BC patients was displayed in Figure 4C.

**Figure 5:**
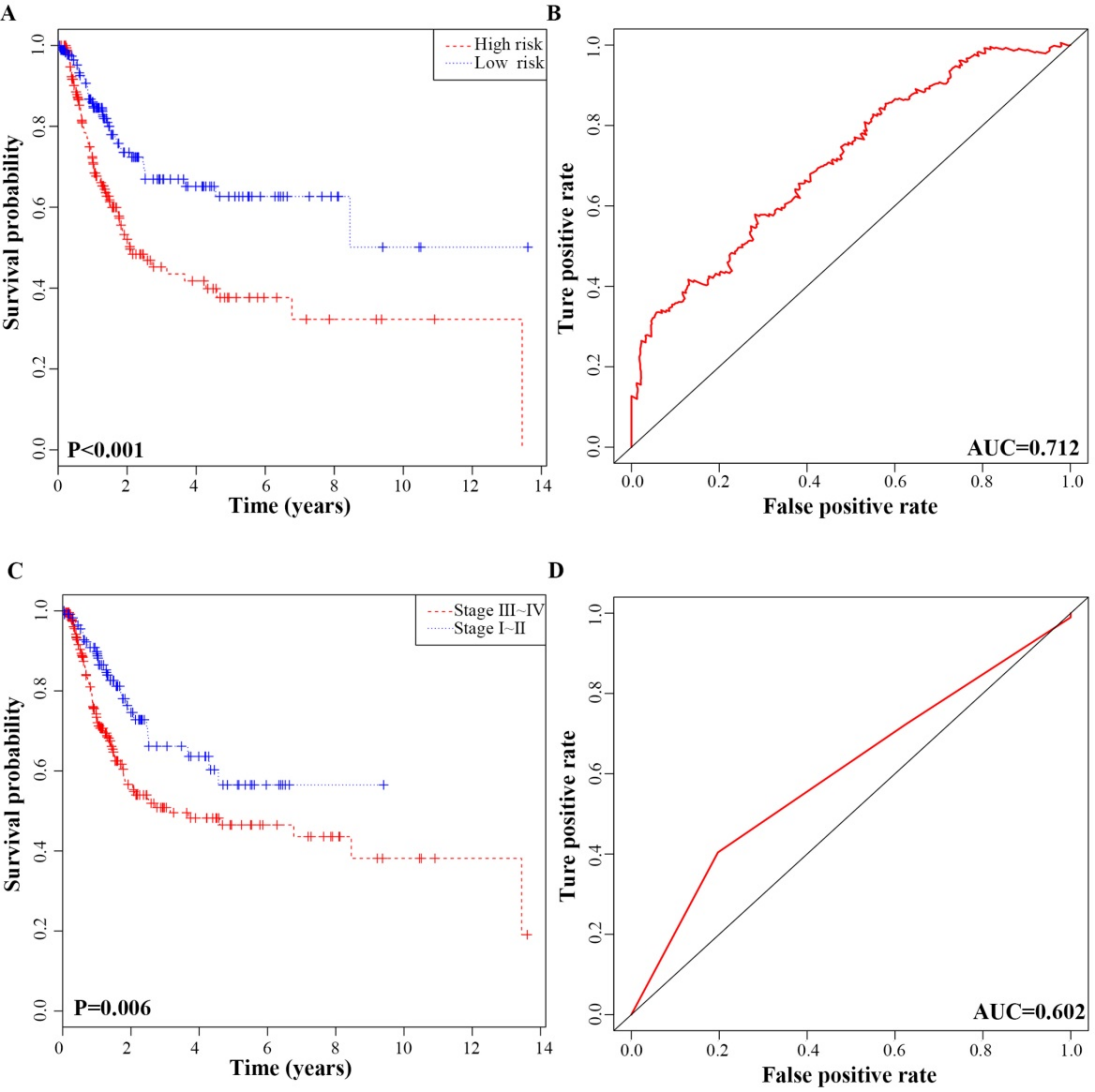
ROC curves and Kaplan-Meier plot based on the integrated classifier in bladder cancer patients. **A,** Kaplan-Meier analysis of recurrence free survival between the high‑risk and low‑risk groups. **B,** Time-dependent ROC curve with AUC of risk score built by the 6-lncRNA signature. **C,** Kaplan-Meier analysis of recurrence free survival between stage I+II and stage III+IV patients **D,** Time-dependent ROC curve analysis of pathological stage.

In addition, we performed K-M analysis, which revealed that the high-risk group was correlated with worse RFS compared to that of the low-risk group (P-value < 0.001; Fig. 5A). In ROC curve analysis, the AUC of the 6-lncRNA signature was 0.712, indicating its utility as a prognostic model for predicting the recurrence status of BC (Fig. 5B). To display the prognostic efficiency of risk score more intuitively, we also generated a K-M plot of tumour pathological stage and the corresponding ROC curve. Tumour pathological stage also divided the BC patients into two groups (Stage Ⅰ + Stage Ⅱ vs. Stage Ⅲ + Stage Ⅳ) with a significant difference in RFS (P-value = 0.006; Fig. 5C). The AUC of the ROC curve based on pathological stage was 0.602 (Fig. 5D). Although pathological stage is commonly used for recurrence prediction in the clinical setting 5, its recurrence prognostic AUC value is limited compared with risk score. Moreover, the AUC of the ROC curve based on age, gender and grade was 0.523, 0.512, 0.51 respectively, which all were lower than risk prediction model based on the 6-lncRNA signature (Fig. S1).

The correlation between clinical parameters and risk level was investigated. Using chi-square test, the risk level was significantly related to M stage (P= 0.031), Grade stage (P =0.001), pathological stage (P=0.002) and recurrence status (P< 0.001) (Table 4).

### Prognostic value of the six‑lncRNA signature in BC

Univariate and multivariate regression models were used to assess the prognostic power of the 6-lncRNA signature. Univariate analysis indicated that risk score, pathological stage, and N stage were significantly correlated with RFS in BC patients (P < 0.01). However, no significant differences were found between the recurrence survival time and age, gender, T stage, M stage and tumour grade. Multivariate analysis indicated that only N stage and risk score were independent prognostic factors of RFS (both P< 0.001, Table 5).

### Clinical feature analysis of recurrent signatures

**Figure 6:**
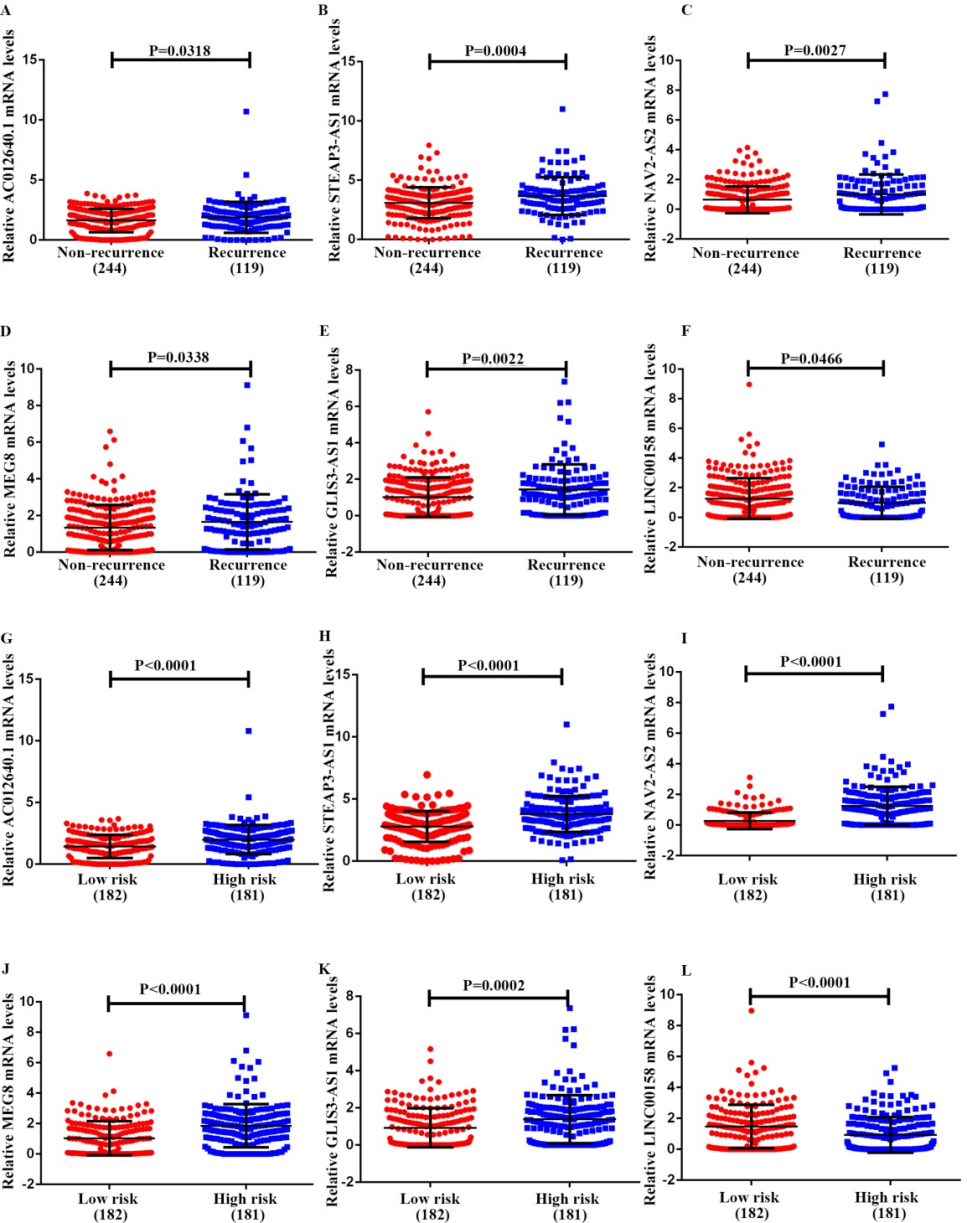
Expression pattern of the 6-lncRNA (AC012640.1, STEAP3-AS1, NAV2-AS2, MEG8, GLIS3-AS1, and LINC00158). **A-F,** recurrence and non-recurrence bladder cancer tissues, and **G-L,** high- risk and low- risk groups.

**Figure 7:**
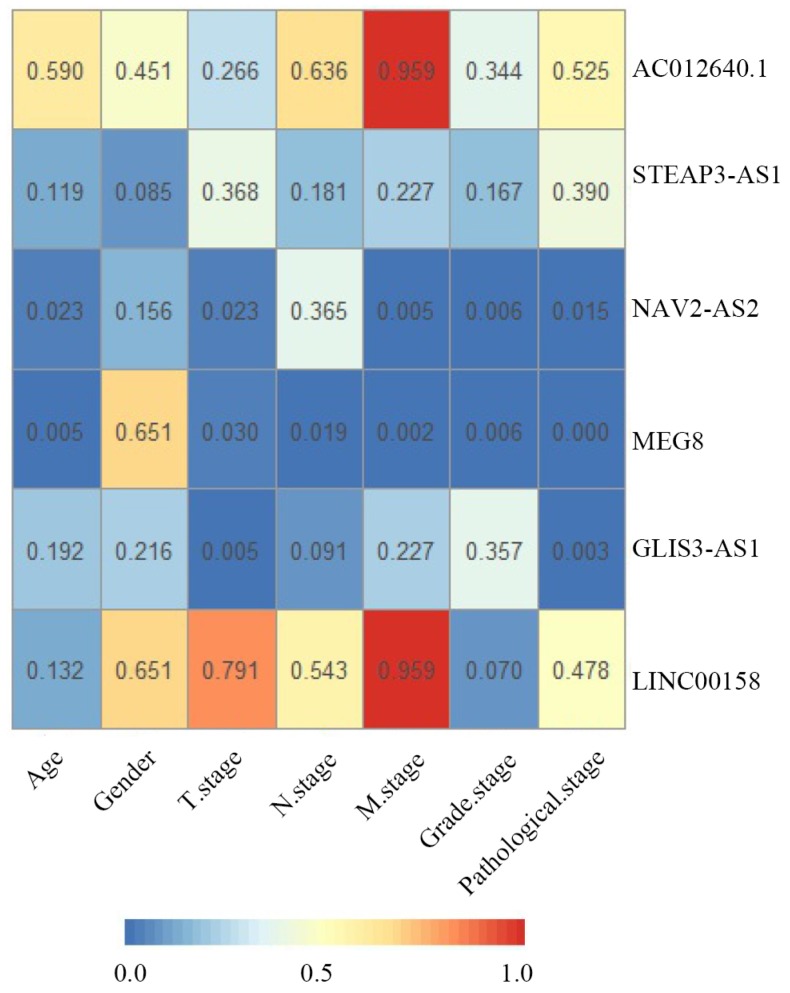
Correlations between clinical parameters and prognostic six signature lncRNAs in bladder cancer. The numbers in each block indicate the P-value. The bluer the block, the smaller the P-value.

To further study the six-lncRNA signatures, we analyzed the expression levels of lncRNAs in non-recurrent and recurrent BC tissues, and in the low- and high-risk patient groups (Fig 6). MEG8, NAV2-AS2, STEAP3-AS1, GLIS3-AS1 and AC012640.1 were up-regulated in patients with a high-risk score, whereas LINC00158 was expressed at high levels in the low-risk patients. Similarly, we found that MEG8, NAV2-AS2, STEAP3-AS1, GLIS3-AS1 and AC012640.1 were expressed at high levels and LINC00158 was expressed at low levels in recurrent BC patients. We also analysed the expression of these lncRNAs under various clinical characteristics and investigated their associations with clinical progress. The results revealed that these genes can be used for effective risk stratification in BC (Table 6, Fig 7).

### Experimental validation

A potential regulatory axis of the STEAP3-AS1/miR-211 in the network was chosen for validation. The relative expression level of STEAP3-AS1 was investigated in four bladder cancer cell lines by qRT-PCR (Fig. S2A). According to the result, BC cell lines T24 and EJ were selected for further experiment. The effects of siRNA knockdown of STEAP3-AS1 in T24 and EJ were examined by qRT-PCR (Fig. S2B-C). Then, the expression level of miR-211 after STEAP3‐AS1 knockdown was also measured by qRT-PCR (Fig. S2D). The miR-211 expression was significantly increased after STEAP3-AS1 knockdown in bladder cancer cell lines.

## Discussion

Bladder cancer is characterized by a high recurrence rate with a variable rate of progression; more than 50% of patients experience recurrence 5,22. It is also the most expensive solid tumour type per patient 6. At present, postoperative follow-up monitoring recurrence of bladder cancer patients mainly relies on cystoscopy. However, the invasiveness of such a procedure limits its use in mass cancer surveillance, and urine cytology is poor at detecting low-grade bladder cancer. Additionally, several non-invasive biomarkers for detecting and predicting the biological behaviour of BC have been identified, such as bladder tumour antigen (BTA), nuclear matrix protein 22 (NMP22) and cytokeratin. These have limited utility in the early examination of BC due to the lack of sensitivity and specificity, and none of them is recommended for large-scale cancer screening 23. Therefore, discovery of effective biomarkers for recurrence prediction of BC can play pivotal roles in predicting of tumour recurrence and assessment recurrence prognosis of BC patients. Growing experimental evidence indicates that lncRNAs play important roles in many biological processes. One tool that can be used to investigate the link between lncRNAs and cancer is ceRNA networks, which are closely related to the development of cancers 16,24-25. Previous studies have revealed several potential ceRNAs in BC, but those studies only evaluated the predictive effect of lncRNAs on the recurrence of non-muscle invasive bladder cancer from a single-centre study 14,26-27. Zhu et al. 28 constructed a lncRNA-related ceRNA network in BC, revealing three key lncRNAs as potential prognostic biomarkers for BC, but they only explored the relationship with overall survival. In terms of recurrence prediction, a lncRNA signature based on the ceRNA network of BC has not been reported.

In our study, we aimed to ascertain whether there are some lncRNAs based on the ceRNA network that may be involved in the recurrence of BC. Using miRNA as a bridge, paired lncRNA-miRNA-mRNA was screened to construct a ceRNA network, providing insight into BC. However, due to the complex pathogenesis during initiation and progression of severe malignancy, a single lncRNA may be an unreliable biomarker to predict tumour recurrence in a timely manner. In this regard, we established a lncRNA prognostic panel based on the theory of the ceRNA network to explore the effect of lncRNAs that are significantly associated with RFS. Univariate and multivariate Cox proportional hazard regression analyses were conducted to successively analyse the prognostic value of differentially expressed lncRNAs that identified six lncRNAs (MEG8, NAV2-AS2, STEAP3-AS1, GLIS3-AS1, LINC00158, AC012640.1) as independent risk factors for recurrence-free survival of bladder cancer patients. According to a cumulative risk score of the six lncRNAs, BC patients were divided into low-risk and high-risk groups. Next, the predictive values of clinical characteristics and the risk score were analysed, which indicated that this 6-lncRNA signature independently predicted RFS in BC patients, and the risk level was significantly related to M stage, Grade stage, pathological stage**,** recurrence status. In addition, tumour stage is a currently known predictor of recurrent bladder cancer 5, and the AUC of the ROC curve based on tumour stage to predict recurrence was 0.602. The AUC of the ROC curve based on the 6-lncRNA signature was 0.712, which was higher than the tumour stage. Importantly, these findings provided several clues that the risk score may be particularly useful for recurrence surveillance.

Accumulating evidences support the involvement of lncRNAs in tumourigenesis and progression in various types of cancers including BC by modulating oncogenic and tumour-suppressing pathways 9. Based on the hypothesis of ceRNA, lncRNAs can be mediated by mRNAs; thus, the specific lncRNAs may also function or concentrate on the potential pathways in a manner similar to mRNAs. Our data showed that the mRNAs associated with CC were extracellular space, intermediate filament, extracellular region, keratin filament and nucleosome. Living cells secrete a large number of endocytic or plasma membrane vesicle, including exosomes, and microvesicles (MVs) into extracellular space, which have great potential applications in cancer diagnosis, prognosis, and epidemiology 29-30. Intermediate filament is critical molecular for cancer cell invasion and extravasation for metastasis 31. Nestin, a class VI intermediate filament protein, is also an independent predictor of cancer-specific survival after radical cystectomy in bladder cancer patients 32. Meanwhile, the mRNAs related to BP were most relevant to cellular protein metabolic process, regulation of ion transmembrane transport and nucleosome assembly. Cancer cells are well-known to require a constant supply of protein, lipid, RNA, and DNA via altered metabolism for accelerated cell proliferation 33. Ion channels expression and function are strongly modified in solid tumours and vascular malformations 34. Growing evidence indicates that SWI/SNF nucleosome remodelling complexes have a widespread role in tumour suppression, as inactivating mutations in several SWI/SNF subunits have recently been identified at a high frequency in a variety of cancers 35. Some pathways that appeared in the KEGG analysis have been previously reported to be associated with cancer. SLE was associated with an increased risk of bladder cancer patients 36, and the development and progression of bladder cancer have a strong association with steroid hormonal pathways 37. Smoking is a definitive cause of bladder cancer, and tobacco-users were significantly associated with a higher risk of bladder cancer 38. These functions and pathways are closely related to the occurrence and development of BC.

In the current study, among this 6-lncRNA signature, NAV2-AS2, STEAP3-AS1 and GLIS3-AS1 are antisense lncRNAs, and there is growing evidence that a large number of antisense lncRNAs play crucial roles in cancer 39-40. MEG8 is located in the Dlk1-Dio3 region, and this region harbours one of the largest microRNA clusters in the human genome, consisting of 54 miRNAs. Aberrant expression of several miRNAs located within this region has been implicated in the pathogenesis of various cancers 41. In our study, we noticed that MEG8 with high-expression could compete with downregulated hsa-miR-506 in the ceRNA network, as well as LINC00158 and GLIS3-AS1. Previous studies have shown that silencing miR-506 expression levels could influence capacity of proliferation, invasion, metastasis and other processes in cancer cells 42-44. High expression of NAV2-AS2 or STEAP3-AS1 could compete with upregulated hsa-miR-122. Some research found that miR-122 promotes cancer cell proliferation, migration, and invasion 45. However, others hold a contrary opinion 46-47. In addition, high expression of STEAP3-AS1 could compete with upregulated hsa-miR-372, hsa-miR-373 and hsa-miR-217. Importantly, hsa-miR-373 was reported to be associated with poor progression-free survival of BC patients 48. Furthermore, high expression of miR-373 has been observed in high-grade muscle invasive bladder cancer (MIBC) 49. In addition, LINC00158 could compete with hsa-miR-375, and hsa-miR-375 was involved in a seven-miRNA panel for the diagnosis and recurrence prediction of BC 15. In the experiment, we found downregulated expression of STEAP3-AS1 by si-STEAP3-AS1 significantly increased miR-211 expression in T24 and EJ cells. These results suggested that the STEAP3-AS1/miR-211 axis plays essential roles in the ceRNA network and conformed to the 'ceRNA theory', which was consistent with the predictions in our network.

No study thus far has reported any association of the 6-lncRNA signature with bladder cancer. This is the first study to show aberrant expression of MEG8, NAV2-AS2, STEAP3-AS1, GLIS3-AS1, LINC00158, AC012640.1 in BC and indicates the potential for prediction of tumour recurrence and assessment of recurrence prognosis in BC patients. In addition, the 6-lncRNA signature based on the ceRNA network will be helpful in future experimental studies.

Although the findings of our study have important clinical implications, the limitations should also be discussed. First, the BC patient information provided by TCGA will need to be confirmed by other experimental methods. Second, several novel lncRNAs with significant clinical relevance in BC need to be explored further to determine the underlying molecular mechanism. Finally, a longer follow-up duration is required to verify our results.

## Conclusions

In summary, this is the first report integrating a ceRNA network with TCGA data to build a lncRNA-related risk score and evaluate the RFS of BC patients. We revealed a 6-lncRNA signature that could be a prognostic predictor for BC patients using bioinformatics analysis from the TCGA database. Additionally, by building a ceRNA network with BC-specific lncRNAs, miRNAs and mRNAs, we clarified the mechanism of BC at the genetic level better and elucidated the relationship between these three RNA species. Our research increases the understanding of the recurrence characteristics of BC and offers novel lncRNAs to help identify patients with a higher risk of BC recurrence, as well as to serve as potential recurrence prognostic biomarkers. This study will help us explore target molecular therapeutic approaches for recurrence prevention and improve the management of BC patients.

## Supplementary Material

Supplementary figures and tables.

## Figures and Tables

**Table 1:** miRNAs in ceRNA network of BC

miRNA	log FC	*P* Value	FDR
hsa-mir-216b	3.333739	5.58E-18	7.59E-16
hsa-mir-373	2.995232	2.19E-12	7.84E-11
hsa-mir-217	2.691103	1.90E-31	4.32E-29
hsa-mir-216a	2.62031	6.75E-22	1.15E-19
hsa-mir-372	2.138423	7.98E-09	2.17E-07
hsa-mir-206	1.711379	3.95E-08	8.96E-07
hsa-mir-375	1.433181	8.06E-08	1.77E-06
hsa-mir-215	1.234865	3.70E-06	5.14E-05
hsa-mir-122	1.226356	3.61E-03	0.022329
hsa-mir-383	-1.08906	2.51E-04	0.002134
hsa-mir-508	-1.53772	7.58E-09	2.15E-07
hsa-mir-506	-1.80214	9.49E-07	1.70E-05
hsa-mir-211	-1.96797	3.39E-07	6.80E-06

Abbreviations: log FC, log2 Fold Change; FDR, False Discovery Rate.

**Table 2:** mRNA in ceRNA network of BC

mRNA	log FC	P Value	FDR
ELAVL4	1.72452395	1.65E-13	1.68E-11
LEFTY1	1.35801541	5.78E-07	2.01E-05
DIO1	1.34487304	1.74E-08	8.07E-07
PRLR	1.04257165	1.06E-05	0.000286

Abbreviations: log FC, log2 Fold Change; FDR, False Discovery Rate.

**Table 3:** lncRNA in ceRNA network of BC

lncRNA	Log FC	*P* Value	FDR
LINC00523	3.657581616	5.04E-13	9.97E-11
ZNF385D-AS2	3.373564484	3.23E-14	7.71E-12
MIR7-3HG	2.944027367	2.47E-14	6.25E-12
SOX2-OT	2.818684005	2.14E-34	1.73E-30
AC012640.1	2.257798517	3.19E-30	1.29E-26
DAPK1-IT1	2.05333515	6.03E-15	1.75E-12
NAV2-AS2	1.718211467	2.16E-10	2.51E-08
AL157387.1	1.539372113	1.78E-03	0.023532
LINC00052	1.464909146	1.64E-03	0.022063
MAGI1-AS1	1.447774408	3.87E-06	0.000144
AL353597.1	1.426748333	1.58E-05	0.000465
STEAP3-AS1	1.377476469	3.64E-16	1.55E-13
LINC00112	1.313437922	1.22E-07	7.24E-06
MEG8	1.304138968	1.32E-08	1.02E-06
AL139002.1	1.249413973	3.22E-03	0.036598
AC011374.1	1.232777194	2.38E-06	9.55E-05
LINC00028	1.090620255	4.27E-05	0.001089
GLIS3-AS1	1.08140406	2.29E-06	9.28E-05
ST7-AS2	1.038526676	2.28E-04	0.004573
ADAMTS9-AS1	-1.021111944	1.89E-05	0.000543
LINC00158	-1.021859061	1.99E-04	0.004113
FAM181A-AS1	-1.153826155	8.28E-04	0.012832
AC092811.1	-1.268412634	7.50E-06	0.000246
LINC00266-1	-1.406229126	7.07E-07	3.31E-05
C7orf71	-1.472361607	5.13E-07	2.46E-05
C12orf77	-1.579343365	1.63E-04	0.0035
SMCR5	-1.634275303	1.75E-09	1.69E-07
RMRP	-2.975144239	1.13E-09	1.18E-07
LINC00458	-4.956073368	1.21E-13	2.59E-11

Abbreviations: log FC, log2 Fold Change; FDR, False Discovery Rate.

**Table 4:** Relationship between risk level and clinicopathological characteristics

Parameter	No.pts	Risk Score (No.)		P value
		Low Risk (n=182)	High Risk (n=181)	
Age (years)	69(34-89)	69(37-86)	68(34-89)	0.559
**Age (years)**				
<60	78	45	33	0.132
≥60	285	137	148	
**Gender**				
Female	92	44	48	0.608
Male	271	138	133	
**T stage**				
T1 + T2	178	93	85	0.491
T3+ T4	179	87	92	
**N stage**				
N0	225	120	105	0.120
N1	138	62	76	
**M stage**				
M0	187	104	83	0.031^*^
M1	176	78	98	
**Grade stage**				
Low grade	20	17	3	0.001^*^
High grade	342	164	178	
**Pathological stage**				
Stage I +Stage II	118	73	45	0.002^*^
Stage III +Stage IV	245	109	136	
**Recurrence Status**				
No	244	144	100	0.000^*^
Yes	119	38	81	

Ab breviations: pts: patients. *Statistically significant.

**Table 5:** Univariate and multivariate Cox regression analysis for the predictive values of clinical features and risk score

Variable	Univariate analysis		Multivariate analysis	
	HR(95%CI)	P value	HR(95%CI)	P value
**Age (years)**		0.279		0.947
<60	Reference		Reference	
≥60	1.281(0.818-2.006)		0.984(0.617-1.570))	
**Gender**		0.798		0.825
Female	Reference		Reference	
Male	1.056(0.694-1.607)		0.952(0.617-1.469)	
**T stage**		0.174		0.846
T1 + T2	Reference		Reference	
T3 + T4	1.290(0.894-1.862)		0.956(0.605-1.510)	
**N stage**		0.000^*^		0.000^*^
N0	Reference		Reference	
N1	2.350(1.637-3.373)		2.075(1.406-3.061)	
**M stage**		0.179		0.608
M0	Reference		Reference	
M1	1.281(0.892-1.838)		1.104(0.757-1.609)	
**Grade stage**		0.201		0.860
Low Grade	Reference		Reference	
High Grade	2.496(0.615-10.140)		1.141(0.264-4.926)	
**Pathological stage**		0.007^*^		0.325
Stage Ⅰ + Stage Ⅱ	Reference		Reference	
Stage Ⅲ + Stage Ⅳ	1.791(1.177-2.726)		1.319(0.760-2.289)	
**Risk score**		0.000^*^		0.000^*^
Low	Reference		Reference	
High	2.527(1.717-3.718)		2.159(1.445-3.227)	

Abbreviations: HR, Hazard ratio; 95%CI, 95% confidence interval; *Statistically significant.

**Table 6:** The relationship between the 6 signature lncRNAs and clinicopathological characteristics

Parameter	No.	AC012640.1 expression		P value	GLIS3-AS1 expression		P value	LINC00158 expression		P value
		Low (n=181)	High (n=182)		Low (n=181)	High (n=18)		Low (n=182)	High (n=181)	
**Age (years)**										
<60	78	41	37	0.59	44	34	0.192	45	33	0.132
≥60	285	140	145		137	148		137	148	
**Gender**										
Female	92	49	43	0.451	51	41	0.216	48	44	0.651
Male	271	132	139		130	141		134	137	
**T stage**										
T1 + T2	178	84	94	0.266	101	77	0.005^*^	88	90	0.791
T3 + T4	179	95	84		75	104		91	88	
**N stage**										
N0	225	110	115	0.636	120	105	0.091	110	115	0.543
N1	138	71	67		61	77		72	66	
**M stage**										
M0	187	93	94	0.959	99	88	0.227	94	93	0.959
M1	176	88	88		82	94		88	88	
**Grade stage**										
Low Grade	20	12	8	0.344	12	8	0.357	14	6	0.07
High Grade	342	168	174		169	173		168	174	
**Pathological stage**										
Stage I+II	118	56	62	0.525	72	46	0.003^*^	56	62	0.478
Stage III+IV	245	125	120		109	136		126	119	

**Parameter**	**No.**	**MEG8 expression**		**P value**	**NAV2-AS2 expression**		**P value**	**STEAP3-AS1 expression**		**P value**
		**Low (n=181)**	**High (n=182)**		**Low (n=182)**	**High (n=181)**		**Low (n=182)**	**High (n=181)**	
**Age (years)**										
<60	78	50	28	0.005^*^	48	30	0.023^*^	33	45	0.119
≥60	285	131	154		134	151		149	136	
**Gender**										
Female	92	44	48	0.651	52	40	0.156	39	53	0.085
Male	271	137	134		130	141		143	128	
**T stage**										
T1 + T2	178	98	80	0.03^*^	100	78	0.023^*^	94	84	0.368
T3 + T4	179	78	101		79	100		86	93	
**N stage**										
N0	225	123	102	0.019^*^	117	108	0.365	119	106	0.181
N1	138	58	80		65	73		63	75	
**M stage**										
M0	187	108	79	0.002^*^	107	80	0.005^*^	88	99	0.227
M1	176	73	103		75	101		94	82	
**Grade stage**										
Low Grade	20	16	4	0.006^*^	16	4	0.006^*^	13	7	0.167
High Grade	342	165	177		166	176		168	174	
**Pathological stage**										
Stage I+II	118	76	42	0^*^	70	48	0.015^*^	63	55	0.39
Stage III+IV	245	105	140		112	133		119	126	

Abbreviations: The six lncRNAs were divided into 'high' and 'low' lncRNA expression group according to the median value; *Statistically significant.

## Abbreviations

BC: bladder cancer
RFS: recurrence free survival
ceRNA: competing endogenous RNA
DEmRNAs: differentially expressed mRNAs
DElncRNAs: differentially expressed lncRNAs
DEmiRNAs: differentially expressed miRNAs
ROC: receive operating characteristic
lncRNAs: long noncoding RNAs
TCGA: The Cancer Genome Atlas
MRE: miRNA response elements
GO: Gene Ontology
KEGG: Kyoto Encyclopedia of Genes and Genomes
CC: cell components
BP: biological processes
MF: molecular functions
MIBC: muscle invasive bladder cancer

## References

[1] Siegel RL, Miller KD, Jemal A (2018). Cancer statistics, 2018. CA: a cancer journal for clinicians.

[2] Ferlay J, Soerjomataram I, Dikshit R (2015). Cancer incidence and mortality worldwide: sources, methods and major patterns in GLOBOCAN 2012. International journal of cancer.

[3] Kamat AM, Hahn NM, Efstathiou JA (2016). Bladder cancer. Lancet.

[4] Christodouleas JP, Baumann BC, He J (2014). Optimizing bladder cancer locoregional failure risk stratification after radical cystectomy using SWOG 8710. Cancer.

[5] Kaufman DS, Shipley WU, Feldman AS (2009). Bladder cancer. Lancet.

[6] Voltaggio L, Cimino-Mathews A, Bishop JA (2016). Current concepts in the diagnosis and pathobiology of intraepithelial neoplasia: A review by organ system. CA: a cancer journal for clinicians.

[7] Lokeshwar VB, Habuchi T, Grossman HB (2005). Bladder tumor markers beyond cytology: International Consensus Panel on bladder tumor markers. Urology.

[8] Ponting CP, Oliver PL, Reik W (2009). Evolution and functions of long noncoding RNAs. Cell.

[9] Huarte M (2015). The emerging role of lncRNAs in cancer. Nature medicine.

[10] Cabili MN, Trapnell C, Goff L (2011). Integrative annotation of human large intergenic noncoding RNAs reveals global properties and specific subclasses. Genes & development.

[11] Du Z, Fei T, Verhaak RG (2013). Integrative genomic analyses reveal clinically relevant long noncoding RNAs in human cancer. Nature structural & molecular biology.

[12] Qi P, Du X (2013). The long non-coding RNAs, a new cancer diagnostic and therapeutic gold mine. Modern pathology: an official journal of the United States and Canadian Academy of Pathology, Inc.

[13] Yang L, Xue Y, Liu J (2017). Long noncoding RNA ASAP1-IT1 promotes cancer stemness and predicts a poor prognosis in patients with bladder cancer. Neoplasma.

[14] Zhang S, Du L, Wang L (2019). Evaluation of serum exosomal LncRNA-based biomarker panel for diagnosis and recurrence prediction of bladder cancer. Journal of cellular and molecular medicine.

[15] Du L, Jiang X, Duan W (2017). Cell-free microRNA expression signatures in urine serve as novel noninvasive biomarkers for diagnosis and recurrence prediction of bladder cancer. Oncotarget.

[16] Salmena L, Poliseno L, Tay Y (2011). A ceRNA hypothesis: the Rosetta Stone of a hidden RNA language?. Cell.

[17] Song X, Cao G, Jing L (2014). Analysing the relationship between lncRNA and protein-coding gene and the role of lncRNA as ceRNA in pulmonary fibrosis. Journal of cellular and molecular medicine.

[18] Karreth FA, Pandolfi PP (2013). ceRNA cross-talk in cancer: when ce-bling rivalries go awry. Cancer discovery.

[19] Zhou M, Wang X, Shi H (2016). Characterization of long non-coding RNA-associated ceRNA network to reveal potential prognostic lncRNA biomarkers in human ovarian cancer. Oncotarget.

[20] An Q, Zhou L, Xu N (2018). Long noncoding RNA FOXD2-AS1 accelerates the gemcitabine-resistance of bladder cancer by sponging miR-143. Biomedicine & pharmacotherapy = Biomedecine & pharmacotherapie.

[21] Sui J, Li YH, Zhang YQ (2016). Integrated analysis of long non-coding RNAassociated ceRNA network reveals potential lncRNA biomarkers in human lung adenocarcinoma. International journal of oncology.

[22] Turo R, Cross W, Whelan P (2012). Bladder cancer. Medicine.

[23] Chao D, Freedland SJ, Pantuck AJ (2001). Bladder cancer 2000: molecular markers for the diagnosis of transitional cell carcinoma. Reviews in urology.

[24] Tang J, Zhuo H, Zhang X (2014). A novel biomarker Linc00974 interacting with KRT19 promotes proliferation and metastasis in hepatocellular carcinoma. Cell death & disease.

[25] Rutnam ZJ, Du WW, Yang W (2014). The pseudogene TUSC2P promotes TUSC2 function by binding multiple microRNAs. Nature communications.

[26] Du L, Duan W, Jiang X (2018). Cell-free lncRNA expression signatures in urine serve as novel non-invasive biomarkers for diagnosis and recurrence prediction of bladder cancer. Journal of cellular and molecular medicine.

[27] Zhang S, Zhong G, He W (2016). lncRNA Up-Regulated in Nonmuscle Invasive Bladder Cancer Facilitates Tumor Growth and Acts as a Negative Prognostic Factor of Recurrence. The Journal of urology.

[28] Zhu N, Hou J, Wu Y (2018). Integrated analysis of a competing endogenous RNA network reveals key lncRNAs as potential prognostic biomarkers for human bladder cancer. Medicine (Baltimore).

[29] Liu YR, Ortiz-Bonilla CJ, Lee YF (2018). Extracellular Vesicles in Bladder Cancer: Biomarkers and Beyond.

[30] Verma M, Lam TK, Hebert E (2015). Extracellular vesicles: potential applications in cancer diagnosis, prognosis, and epidemiology. BMC clinical pathology.

[31] Sutoh Yoneyama M, Hatakeyama S, Habuchi T (2014). Vimentin intermediate filament and plectin provide a scaffold for invadopodia, facilitating cancer cell invasion and extravasation for metastasis. European journal of cell biology.

[32] Tabata K, Matsumoto K, Minami S (2014). Nestin is an independent predictor of cancer-specific survival after radical cystectomy in patients with urothelial carcinoma of the bladder. PloS one.

[33] Wu P, Liu S, Su J (2017). Apoptosis triggered by isoquercitrin in bladder cancer cells by activating the AMPK-activated protein kinase pathway. Food & function.

[34] Biasiotta A, D'Arcangelo D, Passarelli F (2016). Ion channels expression and function are strongly modified in solid tumors and vascular malformations. Journal of translational medicine.

[35] Wilson BG, Roberts CW (2011). SWI/SNF nucleosome remodellers and cancer. Nature reviews. Cancer.

[36] Song L, Wang Y, Zhang J (2018). The risks of cancer development in systemic lupus erythematosus (SLE) patients: a systematic review and meta-analysis. Arthritis research & therapy.

[37] Godoy G, Gakis G, Smith CL (2016). Effects of Androgen and Estrogen Receptor Signaling Pathways on Bladder Cancer Initiation and Progression. Bladder cancer.

[38] Srivastava DS, Mandhani A, Mittal RD (2008). Genetic polymorphisms of cytochrome P450 CYP1A1 (*2A) and microsomal epoxide hydrolase gene, interactions with tobacco-users, and susceptibility to bladder cancer: a study from North India. Archives of toxicology.

[39] Li T, Xie J, Shen C (2016). Upregulation of long noncoding RNA ZEB1-AS1 promotes tumor metastasis and predicts poor prognosis in hepatocellular carcinoma. Oncogene.

[40] Yuan SX, Tao QF, Wang J (2014). Antisense long non-coding RNA PCNA-AS1 promotes tumor growth by regulating proliferating cell nuclear antigen in hepatocellular carcinoma. Cancer letters.

[41] Benetatos L, Hatzimichael E, Londin E (2013). The microRNAs within the DLK1-DIO3 genomic region: involvement in disease pathogenesis. Cellular and molecular life sciences: CMLS.

[42] Ruiz AJ, Russell SJ (2015). MicroRNAs and oncolytic viruses. Current opinion in virology.

[43] Wang Z, Si M, Yang N (2018). MicroRNA-506 suppresses invasiveness and metastasis of human hepatocellular carcinoma cells by targeting IL8. American journal of cancer research.

[44] Wang XX, Guo GC, Qian XK (2018). miR-506 attenuates methylation of lncRNA MEG3 to inhibit migration and invasion of breast cancer cell lines via targeting SP1 and SP3. Cancer cell international.

[45] Wang Z, Qin C, Zhang J (2017). MiR-122 promotes renal cancer cell proliferation by targeting Sprouty2. Tumour biology: the journal of the International Society for Oncodevelopmental Biology and Medicine.

[46] Wang Y, Xing QF, Liu XQ (2016). MiR-122 targets VEGFC in bladder cancer to inhibit tumor growth and angiogenesis. American journal of translational research.

[47] Guo L, Yin M, Wang Y (2018). CREB1, a direct target of miR-122, promotes cell proliferation and invasion in bladder cancer. Oncology letters.

[48] Bellmunt J, Zhou CW, Mullane SA (2016). Association of tumour microRNA profiling with outcomes in patients with advanced urothelial carcinoma receiving first-line platinum-based chemotherapy. British journal of cancer.

[49] Guancial EA, Bellmunt J, Yeh S (2014). The evolving understanding of microRNA in bladder cancer. Urologic oncology.

