# Supplementary material for: Identification of a six-lncRNA signature based on a competing endogenous RNA network for predicting the risk of tumour recurrence in bladder cancer patients
Source: J Cancer. 2020 Jan 1;11(1):108–20. doi: 10.7150/jca.35801 (PMC6930402; doi:10.7150/jca.35801)
Supplement: Supplementary file 1 — Supplementary figures and tables. [file jcav11p0108s1.pdf]

Table S1 miRNAs targeting lncRNAs of bladder cancer.

lncRNA	miRNA
AC011374.1	hsa-mir-372, hsa-mir-373
AC012640.1	hsa-mir-216b, hsa-mir-383
AC092811.1	hsa-mir-211
ADAMTS9-AS1	hsa-mir-216a
AL139002.1	hsa-mir-215
AL157387.1	hsa-mir-375
AL353597.1	hsa-mir-215, hsa-mir-216b
C12orf77	hsa-mir-372, hsa-mir-373, hsa-mir-215, hsa-mir-206, hsa-mir-211, hsa-mir-216b
C7orf71	hsa-mir-217, hsa-mir-508
DAPK1-IT1	hsa-mir-215, hsa-mir-206, hsa-mir-211
FAM181A-AS1	hsa-mir-206, hsa-mir-211, hsa-mir-216b, hsa-mir-508
GLIS3-AS1	hsa-mir-206, hsa-mir-506
LINC00028	hsa-mir-211, hsa-mir-216a, hsa-mir-383
LINC00052	hsa-mir-216b, hsa-mir-217
LINC00112	hsa-mir-508, hsa-mir-122
LINC00158	hsa-mir-206, hsa-mir-211, hsa-mir-375
LINC00266-1	hsa-mir-217
LINC00458	hsa-mir-211, hsa-mir-122, hsa-mir-506, hsa-mir-375, hsa-mir-383
LINC00523	hsa-mir-215, hsa-mir-217, hsa-mir-508, hsa-mir-122
MAGI1-AS1	hsa-mir-508
MEG8	hsa-mir-206, hsa-mir-506
MIR7-3HG	hsa-mir-211
NAV2-AS2	hsa-mir-211, hsa-mir-122
RMRP	hsa-mir-206, hsa-mir-122
SMCR5	hsa-mir-372, hsa-mir-373, hsa-mir-215, hsa-mir-206, hsa-mir-211
SOX2-OT	hsa-mir-215, hsa-mir-206, hsa-mir-211, hsa-mir-216b, hsa-mir-508, hsa-mir-122, hsa-mir-506, hsa-mir-375
ST7-AS2	hsa-mir-215, hsa-mir-206, hsa-mir-211, hsa-mir-216b, hsa-mir-122
STEAP3-AS1	hsa-mir-372, hsa-mir-373, hsa-mir-215, hsa-mir-206, hsa-mir-211, hsa-mir-217, hsa-mir-122, hsa-mir-506, hsa-mir-383
ZNF385D-AS2	hsa-mir-375

Table S2 miRNAs targeting mRNAs of bladder cancer

miRNA	mRNA
hsa-mir-372	LEFTY1
hsa-mir-383	DIO1
hsa-mir-375	ELAVL4
hsa-mir-211	PRLR
hsa-mir-373	LEFTY1

Table S3 Prognostic value of the lncRNAs analyzed by univariate Cox's proportional hazards regression model

lncRNA	HR	z value	P value
NAV2-AS2	1.29341153	3.901678729	9.55E-05
STEAP3-AS1	1.267430559	3.810852441	0.000138488
MEG8	1.237899498	2.822528236	0.004764663
MIR7-3HG	1.214849845	2.608994995	0.009080857
LINC00523	1.292727782	2.397639115	0.016501114
GLIS3-AS1	1.176873861	2.339809931	0.019293556
LINC00158	0.84189107	-2.284017853	0.022370482
AL139002.1	1.08961223	2.171480535	0.029894867
AC012640.1	1.222815845	2.113442353	0.034562924

Abbreviations: log FC, log2 Fold Change; FDR, False Discovery Rate

Figure S1

A

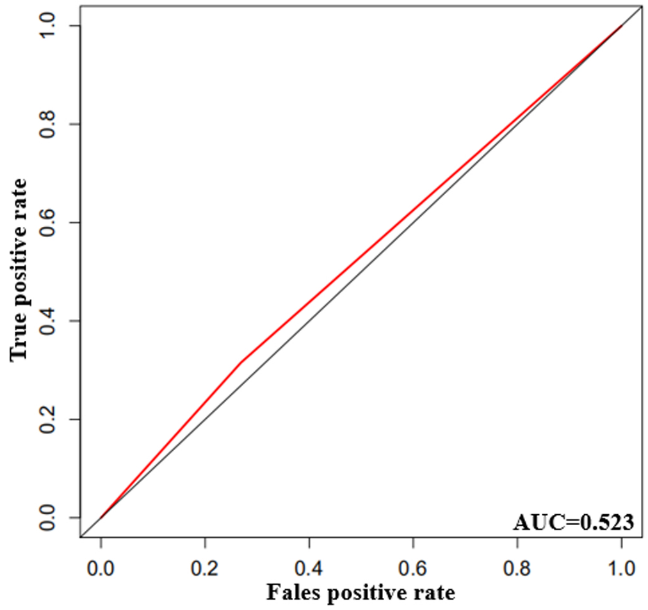

B

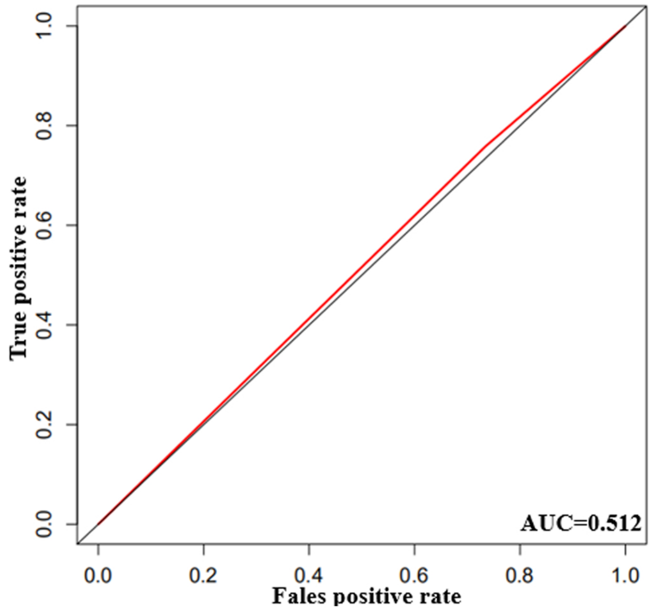

C

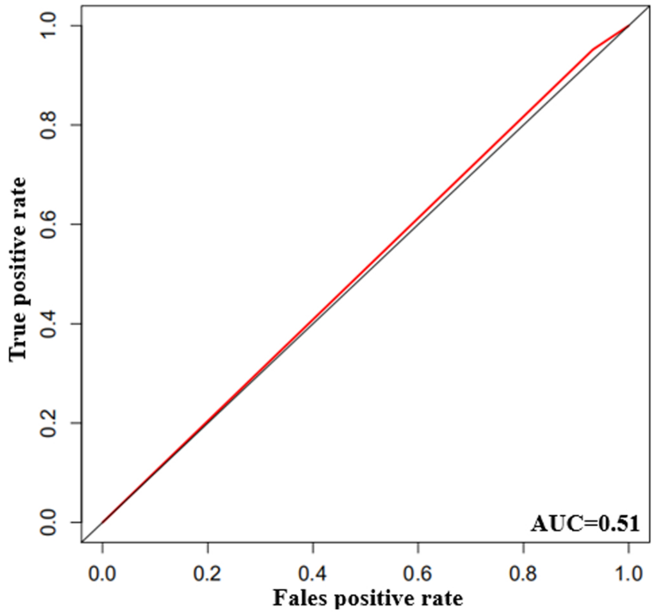

Figure S2

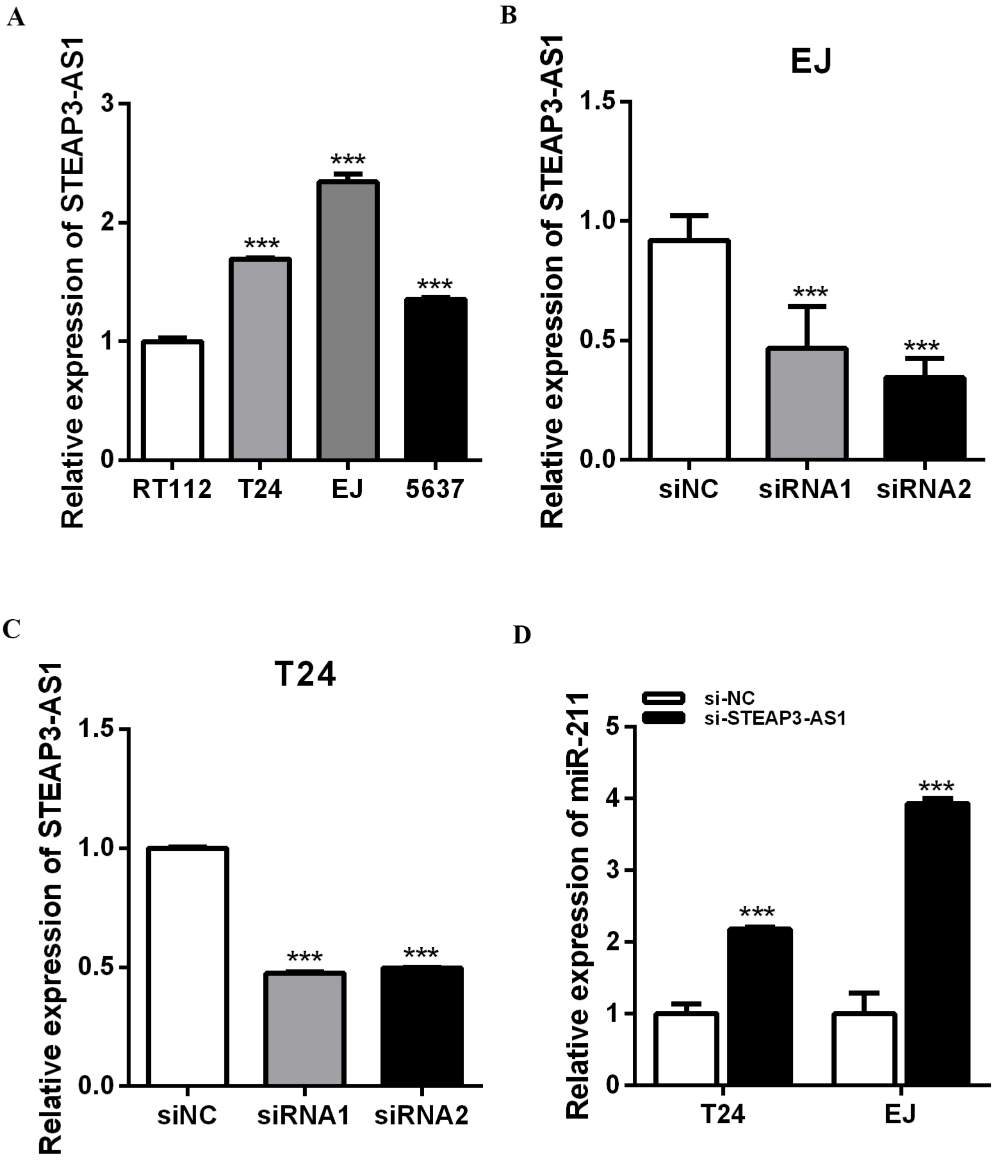
A
B
EJ
C
T24
D
Relative expression of STEAP3-AS1
RT112
T24
EJ
5637
siNC
siRNA1
siRNA2
si-NC
si-STEAP3-AS1
Relative expression of miR-211
***
